# Short-term recovery in patients suffering hypoparathyroid after thyroidectomy: a case control study

**DOI:** 10.1186/s12893-021-01173-8

**Published:** 2021-04-21

**Authors:** Duntao Su, Fada Xia, Wanze Huang, Zhejia Zhang, Ning Bai, Di Wang, Xin Liao, Xinying Li

**Affiliations:** Department of General Surgery, Xiangya Hospital, Central South University, 87 Xiangya Road, Changsha, 410008 China

**Keywords:** Hypoparathyroidism, Hypocalcemia, Thyroidectomy

## Abstract

**Background:**

Postoperative hypoparathyroidism is the main reason for outpatient follow-up and long-term oral calcium and calcitriol treatment. Our study investigated the influencing factors and powerful predictors of short-term postoperative parathyroid function recovery.

**Methods:**

Logistic regression was used to compare the clinicopathological characteristics; surgical details; and serum calcium (Ca), magnesium (Mg), and phosphorus (P) concentrations of patients. A receiver operating characteristic (ROC) curve was used to analyze the predictors of normal parathyroid hormone (PTH).

**Results:**

Among the 111 patients with PTH < 10 pg/mL on the first postoperative day, most patients experienced a return to normal PTH (PTH > 15 pg/mL) within 30 days postoperatively. Univariate analysis showed that Pod (postoperative day) 1 PTH, Pod3 PTH, Pod7 Ca, Pod7 Mg, and Pod7 P (P < 0.05) were associated with parathyroid function recovery to normal on the seventh postoperative day. Multivariate logistic regression analysis revealed the following independent risk factors for normal PTH levels at Pod7 after thyroidectomy: Pod3 PTH (P = 0.038), Pod1 PTH (P = 0.056), Pod7 Mg (P = 0.001), Pod7 P (P = 0.020), and the number of parathyroid glands in situ intraoperatively. The combined sensitivity of serum magnesium concentration and phosphorus concentration to predict parathyroid function recover to normal on the seventh postoperative day was 82.76%, with a sensitivity of 76.83%.

**Conclusion:**

Serum magnesium, phosphorus and PTH concentrations are important influencing factors and effective predictors of short-term postoperative parathyroid function recovery to normal. Serum ion is an effective auxiliary diagnostic method for hypoparathyroidism after thyroidectomy.

## Background

Hypoparathyroidism is one of the most common complications after total thyroidectomy [1–3]. Accidental resection, thermal injury, and devascularization of the parathyroid glands are generally considered the common causes of hypoparathyroidism after thyroid surgery [4]. The need for frequent postoperative monitoring of serum ions and parathyroid function concentration, as well as regular adjustment of the dosage of calcium and calcitriol, increase the financial burden of these patients [5]. Studies have shown that patients with PTH < 10 pg/ml on the morning of the first day after surgery are recommended to extend the length of hospital stay and supplement calcium and vitamin D, while patients with PTH > 10 pg/ml can be safely discharged early [6]. There is also literature suggesting that PTH > 10 pg/ml in the short term after thyroid surgery can reduce the length of hospital stay [7]. Therefore, we chose < 10 pg/ml as the upper limit. Calcium supplementation and calcitriol can effectively prevent the occurrence of hypocalcemia and promote the recovery of parathyroid function [8, 9]. However, these treatments can cause gastrointestinal discomforts, such as constipation in the short term, and increase the risk of kidney stones, gout, hypercalcemia, hypercalciuria, and hyperphosphatemia [10]. Thus, high-frequency follow-up of patients treated with calcitriol and calcium is necessary.

The follow-up visit on Pod7 is the first reexamination after the patients return to their social life after discharge from the hospital. The patients and doctors pay more attention to the results, and the patients are also anxious. Previous studies have measured PTH levels on day 7 after surgery and suggested that protracted hypoparathyroidism recovery mainly depends on the dose of calcium salt and calcitriol at discharge. In the first weeks after thyroidectomy, high-dose calcium and calcitriol supplementation can help the recovery of parathyroid function [9]. A retrospective large study showed that the preoperative PTH level, the percentage of PTH reduction after the operation, and diabetes are influencing factors for the recovery of parathyroid function one week after discharge [11]. However, there are few studies on the short-term parathyroid function return to normal after surgery in patients with hypoparathyroidism. Moreover, there is currently no consensus regarding the appropriate follow-up frequency and biochemical measures. Therefore, the main purpose of this study was to investigate effective ionized predictors and influencing factors of short-term parathyroid function recovery to normal after thyroidectomy.

## Methods

This was a retrospective study of patients undergoing thyroid surgery from January 2018 to May 2019 at the Department of Thyroid Surgery, Xiangya Hospital, Central South University. To ensure the homogeneity of all samples, according to the inclusion criteria and exclusion criteria, we collected enough cases in 16 months. This study was approved and agreed upon by the Ethics Committee of Xiangya Hospital of Central South University, and all patients participating in the study provided written informed consent. All operations were performed by the same experienced surgical team. The inclusion criteria were patients with PTH levels < 10 pg/mL on the first day after thyroidectomy who could complete follow-up after surgery. The exclusion criteria were patients with a previous history of thyroid or parathyroid surgery, parathyroid disease, MEN I or II, abnormal albumin levels before or after surgery, and chronic or acute renal insufficiency. In total, 111 patients were ultimately included in the study.

### Patients' clinical data

The demographic information collected from the patients included sex and age. The data collected related to the operation included the scope of the operation, the number of parathyroid glands retained in situ (PGRIS) during the operation, and the postoperative pathological results of the thyroid nodules. Biochemical indicators, including preoperative serum calcium, magnesium, phosphorus, and 25-hydroxyvitamin D3 levels; PTH levels; and serum calcium, magnesium, and phosphorus levels were measured on days 1, 3, 7, and 30 after surgery.

### Preoperative preparation and parathyroid gland characterization

Examinations included laryngoscopy and ultrasonography of the thyroid and neck lymph nodes before surgery. Thyroid function, PTH, albumin, and serum ions were measured within one week before surgery. The surgical indications for total thyroidectomy were as follows: 1. benign nodules occupying the entire thyroid or almost no normal thyroid tissue; 2. multiple malignant nodules on both sides of the thyroid, with a single tumor diameter of > 1 cm, and malignant tissues with extraglandular invasion requiring central lymph node dissection (CND); and 3 patients with lateral lymph node dissection (LND) who were highly suspected of cervical lateral lymph node metastasis by FNAB, CT, and ultrasound examination. All patients defaulted to having 4 parathyroid glands in the normal position of the parathyroid gland. During the operation, the surgeon did not intentionally search for ectopic parathyroid glands and recorded the number of PGRIS. Intraoperative parathyroid glands are based on visual recognition, and any suspicious parathyroid tissues were sent for intraoperative frozen section pathological biopsy. The number of PGRIS during the operation was divided into 5 groups: group 1, no PGRIS; group 2, 1 PGRIS; group 3, 2 PGRIS; group 4, 3 PGRIS; and group 5, 4 PGRIS. The parathyroid glands that were accidentally removed or had obvious color changes during the operation were excised and preserved in 0.9% saline. A thorough examination was performed to find any inadvertently left parathyroid glands in the specimen after total thyroidectomy. Parathyroid autoimplantation was performed by cutting the parathyroid glands into 1-mm-sized pieces and placing them into the ipsilateral sternocleidomastoid muscle.

### Postoperative management and follow-up

The patients prophylactically received intravenous infusion of calcium gluconate (0–2 g/day) from the first postoperative day as well as oral calcium (1.2–3.6 g/day) and calcitriol (0.25–0.75 μg/day). The dose at which patients did not develop symptomatic hypocalcemia was considered adequate. If patients developed symptomatic hypocalcemia upon stopping intravenous calcium supplement, intravenous calcium was supplement again. All patients were discharged on the third day after surgery. Our hospital informed these patients to follow up on the seventh day and one month after surgery so that the parathyroid function return to normal and serum ion concentrations could be measured and the doses of calcium and calcitriol be adjusted. The main purpose of adjustment is to prevent the patients from symptomatic hypocalcemia. If PTH and serum ions were found to be lower than the normal range in the outpatient follow-up, it was suggested that patients increase or maintain the existing dose. If they were in the normal range, it was recommended that the patient reduce one tablet of calcium per day per week. If symptomatic hypocalcemia occurred after reduction, the original dose should be maintained. Thereafter, the same surgical team followed these patients for at least one year at 3-month intervals.

### Definitions

The symptoms and signs of symptomatic hypocalcemia include numbness or tingling around the mouth and fingers, muscle spasms, or cramps in the limbs. Other signs include the Chvostek sign and Trousseau sign. PTH was measured using an electrochemiluminescence immunoassay (Roche Elecsys System, Roche Diagnostics, Mannheim, Germany), and serum calcium was measured by the o-cresolphthalein complexone method (reference values 15–65 pg/mL and 2.0–2.6 mmol/L, respectively, according to the manufacturer’s normative data). Hypoparathyroidism was defined as PTH level below the normal range. PTH < 15 pg/mL has been adopted by some studies [6, 12, 13]. Normal parathyroid function was defined as PTH > 15 pg/mL without oral calcium or calcitriol replacement therapy. Permanent hypoparathyroidism was defined as postoperative PTH < 15 pg/mL lasting for more than 1 year and requiring oral calcium or calcitriol replacement therapy. The serum magnesium concentration reference value ranges from 0.66 to 1.07 mmol/L, and the serum phosphorus concentration value reference ranges from 0.86 to 1.78 mmol/L.

### Statistical analysis

According to the recovery trend of serum ions and PTH, linear interpolation and the linear trend of neighboring points were used for variables with missing values. These variables included the Pod3 PTH (2.7%), Pod3 Mg (1.8%), Pod3 Ca (1.8%), and Pod3 P (1.8%); Pod7 PTH (1.8%), Pod7 Mg (5.4%), Pod7 Ca (4.5%), and Pod7 P (5.4%); and Pod30 PTH (4.5%). The optimal cut-off values for serum phosphorus concentration, serum calcium concentration, and serum magnesium concentration on the seventh postoperative day and for PTH on the first and third postoperative days were determined by receiver operating characteristic (ROC) curve analysis. This analysis method uses the maximum value of the Youden index as the cut-off value [11]. The area under the ROC curve represents the predictive power of laboratory parameters. Categorical variables are represented by frequencies, and the data of continuous variables conforming to a normal distribution are expressed as the means ± standard deviation. If the data did not conform to a normal distribution, then they were expressed by the medians and IQR. Continuous variables were compared using the Kruskal–Wallis test to examine the differences between groups, and categorical variables were compared using the chi-square test. If there were theoretical numbers < 10 in the categorical variables, then Fisher's exact probability test was used to obtain the p-value. Binomial logistic regression analysis with predictors selected by a forward stepwise procedure was used to assess the predictive factors for hypoparathyroidism on Pod7. Statistical analysis was performed using SPSS 25.0, Medcalc 15.8, and Excel 2019. All tests were two-tailed tests, and *P* < 0.05 was considered statistically significant.

## Results

Of the 111 patients with PTH < 10 pg/mL on the first day after total thyroidectomy, 92 (82.88%) were female, with an average age of 41 years. Among them, 106 (95.50%) patients had papillary thyroid cancer, 65 (58.56%) patients underwent bilateral total thyroidectomy + CND, and 43 (38.74%) patients underwent LND. On average, each patient had 3.0 parathyroid glands retained; 5 (4.50%) patients did not have any parathyroid glands retained during surgery, and 43 (38.74%) patients had all 4 parathyroid glands retained in situ. No iatrogenic hypercalcemia was found during our follow-up. The specific study population characteristics are listed in Table 1

**Table 1:** Characteristics of the study population (N = 111)

Characteristics	n	Value
Age, years	111	41 (32–51)
Sex	111	
Male	19 (17.12%)	NA
Female	92 (82.88%)	NA
Vitamin D (ng/mL)	111	21.10 (16.52–27.41)
Hyperthyroidism		
Yes	1 (0.90%)	NA
No	110 (99.10%)	NA
Scope of surgery		
TT	3 (2.70%)	NA
TT + CND	65 (58.56%)	NA
TT + CND + LND	43 (38.74%)	NA
Thyroid pathology		
Goiter	5 (4.50%)	NA
Carcinoma	106 (95.50%)	NA
PGRIS		
0	5 (4.50%)	NA
1	5 (4.50%)	NA
2	19 (17.12%)	NA
3	39 (35.14%)	NA
4	43 (38.74%)	NA
Preoperative PTH (pg/mL)	111	39.27 (16.52–27.41)
Preoperative Ca (mmol/L)	111	2.35 (0.09)
Preoperative Mg (mmol/L)	111	0.89 (0.84–0.92)
Preoperative P (mmol/L)	111	1.17 (0.19)
Characteristics at postoperative day 1		
Pod1 PTH (pg/mL)	111	5.13 (3.70–6.97)
Pod1 Ca (mmol/L)	111	1.98 (0.12)
Pod1 Mg (mmol/L)	111	0.69 (0.06)
Pod1 P (mmol/L)	111	1.54 (1.40–1.72)
Characteristics at postoperative day 3		
Pod3 PTH (pg/mL)	111	4.91 (3.56–7.02)
Pod3 Ca (mmol/L)	111	1.89 (1.83–2.00)
Pod3 Mg (mmol/L)	111	0.67 (0.63–0.71)
Pod3 P (mmol/L)	111	1.67 (0.34)
Characteristics at postoperative day 7		
Pod7 PTH (pg/mL)	111	10.96 (6.42–15.38)
Pod7 Ca (mmol/L)		
All	111	2.12 (0.19)
Pod7 Ca ≥ 2.05 (mmol/L)	75 (67.57%)	2.23 (2.30–2.14)
Pod7 Ca < 2.05 (mmol/L)	36 (32.43%)	1.94 (1.98–1.84)
Pod7 Mg (mmol/L)		
All	111	0.80 (0.09)
Pod7 Mg > 0.8 (mmol/L)	54 (48.65%)	0.85 (0.91–0.83)
Pod7 Mg ≤ 0.8 (mmol/L)	57 (51.35%)	0.75 (0.77–0.70)
Pod7 P (mmol/L)		
All	111	1.54 (0.29)
Pod7 P ≤ 1.51 (mmol/L)	57 (51.35%)	1.36 (1.45–1.20)
Pod7 P > 1.51 (mmol/L)	54 (48.65%)	1.73 (1.87–1.63)
Characteristics at postoperative day 30		
Pod30 PTH (pg/mL)	111	30.44 (20.38–42.41)
Pod30 Ca (mmol/L)	111	2.25 (2.11–2.33)
Pod30 Mg (mmol/L)	111	0.86 (0.80–0.89)
Pod30 P (mmol/L)	111	1.26 (1.09–1.42)
BMI	111	23 (3.1)
Operation time (min)	111	146(77.9)
Diabetes mellitus		
Yes	1(99.1%)	NA
No	110(0.9%)	NA

Data are presented as the n/N (%), means (SD), and medians (IQR)

*TT* total thyroidectomy, *CND* central lymph node dissection, *LND* lateral lymph node dissection, *PGRIS* parathyroid glands remaining in situ, *Pod* postoperative day

### Parathyroid function return to normal and the changing trend of serum ions within 1 month after thyroidectomy

**Fig. 1:**
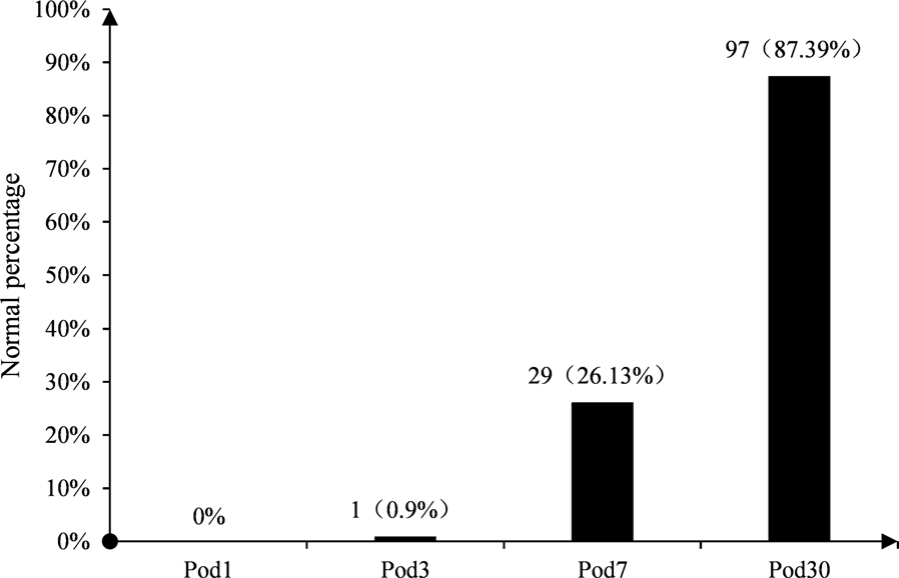
PTH returns to normal on days 1, 3, 7, and 30 after surgery. *Pod* postoperative day

**Fig. 2:**
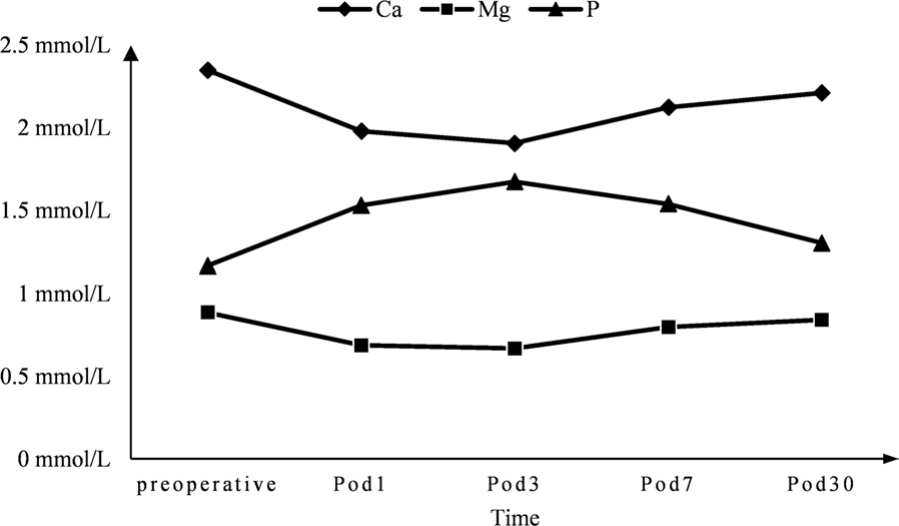
Changing trend in serum ionized Ca, Mg and P before and after thyroidectomy. *Pod* postoperative day

In this study, parathyroid function returned to normal on the third postoperative day in only one patient (0.90%), while it returned to normal on the seventh postoperative day in 29 patients (26.13%) and on the thirtieth postoperative day in 97 patients (87.39%). Most of the patients did not return to normal PTH on Pod7, but most recovered to normal PTH on the first month after thyroidectomy. The recovery of patients with hypoparathyroidism after surgery is shown in Fig. 1. The changing trends in serum Ca, Mg, and P concentrations before and after surgery are shown in Fig. 2.

### The predictive effect of serum Mg concentration and serum P concentration on parathyroid function recovery to normal on the seventh postoperative day

**Fig. 3:**
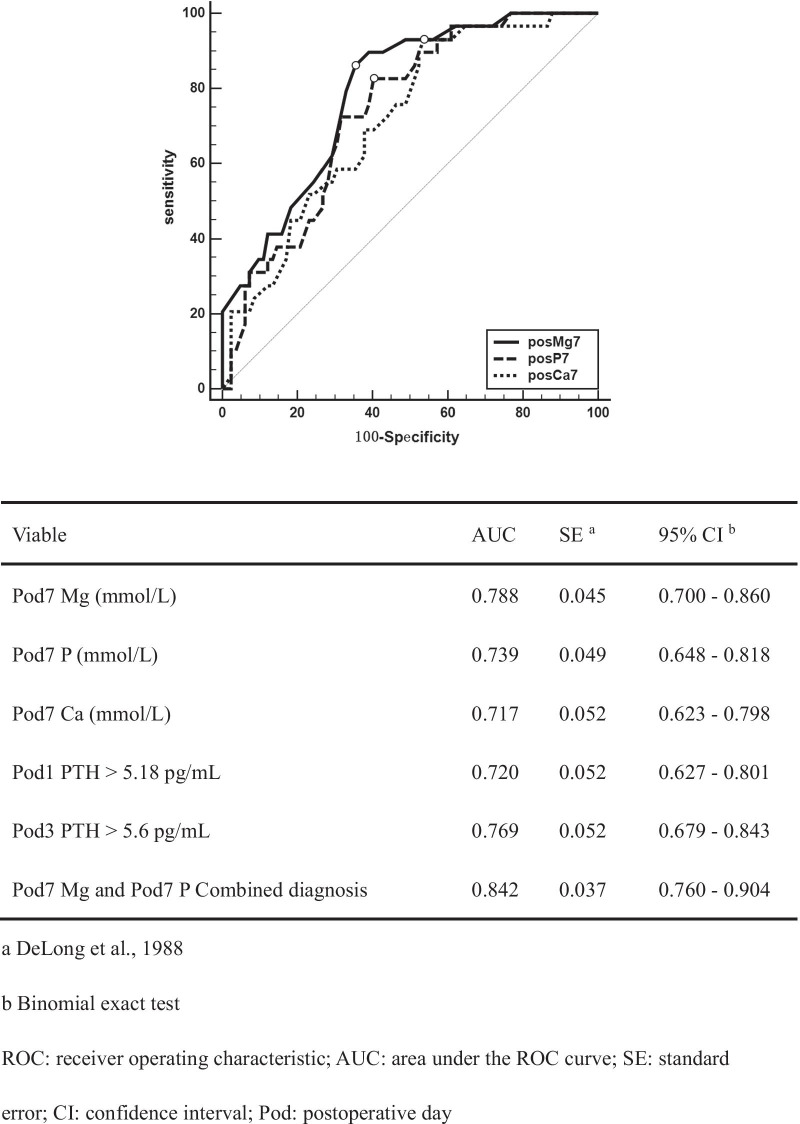
ROC curves corresponding to serum ion concentrations

The predictive effect of Pod7 Mg and Pod7 P on parathyroid function recovery to normal on the seventh postoperative day was determined using ROC curve analysis, and the results are shown in Fig. 3. Both had good diagnostic efficiency for normal PTH. The optimal cut-off values for serum ions on the seventh postoperative day and blood PTH levels on the first and third postoperative days were Pod7 Mg > 0.8 mmol/L, Pod7 P ≤ 1.51 mmol/L, Pod7 Ca > 2.05 mmol/L, Pod1 PTH > 5.18 pg/mL, and Pod7 PTH > 5.6 pg/mL. The combined sensitivity and specificity of serum Mg and P concentrations to predict normal PTH on the seventh day after total thyroidectomy were 82.76% and 76.83%, respectively. This was superior to the diagnostic performance of Pod7 Mg, Pod7 P, Pod1 PTH, or Pod3 PTH alone (Table 2).

**Table 2:** The diagnostic sensitivity, specificity, positive predictive value and negative predictive value of the cut-off values of serum ionized calcium, magnesium and phosphorus for normal PTH levels at Pod7

	Sensitivity (%)	Specificity (%)	PPV (%)	NPV (%)
Pod7 Mg > 0.80 mmol/L	86.21	64.6	46.3	93
Pod7 P ≤ 1.51 mmol/L	82.76	59.76	42.1	90.7
Pod7 Ca > 2.05 mmol/L	93.10	46.34	46.2	84.9
Pod1 PTH > 5.18 pg/mL	79.31	62.20	42.6	89.5
Pod3 PTH > 5.60 pg/mL	75.86	71.95	48.9	89.4
Pod7 Mg and Pod7 P Combined diagnose	82.76	76.83	55.8	92.6

*PPV* positive predictive value, *NPV* negative predictive value, *Pod* postoperative day

### Influencing factors of parathyroid function recovery to normal on the seventh postoperative day

The seventh day after operation is a relatively concerning time for reexamination. Univariate analysis showed that Pod1 PTH (P < 0.05), Pod3 PTH (P < 0.05), Pod7 Mg (P < 0.05), Pod7 Ca (P < 0.05), and Pod7 P (P < 0.05) were the influencing factors of parathyroid function recovery to normal on the seventh day after surgery (Table 3). However, the scope of surgery was not a factor influencing the return of PTH to normal after surgery. Intraoperative PGRIS (OR 0.025 P = 0.025), Pod3 PTH > 5.6 pg/mL (OR 3.745 P = 0.038), Pod1 PTH > 5.18 pg/mL (OR 3.439 P = 0.056), Pod7 Mg ≥ 0.8 mmol/L (OR 19.221 P = 0.001) and Pod7 P ≤ 1.51 mmol/L (OR 4.475 P = 0.02) were included in the final multifactor analysis equation, and the results are shown in Table 4.

**Table 3:** Univariate analysis for Pos7 PTH

Covariate	OR	95%CI	*P* value
Age, years (mean ± SD)	1.01	(0.98, 1.05)	0.531
Continuous sex	1.01	(0.33, 3.11)	0.984
Female	Reference		
Male	1.01	(0.33, 3.11)	0.984
Vitamin D	0.98	(0.93, 1.04)	0.541
BMI	0.93	(0.80,1.07)	0.925
Operation time	1.00	(0.99,1.01)	0.991
Diabetes mellitus	4,731,033,468	(0.00, NA)	1.00
Continuous scope of surgery	0.93	(0.42, 2.06)	0.855
TT	Reference		
TT + CND	6,464,692.49	(0.00, Inf)	0.991
TT + CND + LND	4,742,836.60	(0.00, Inf)	0.991
Continuous PGRIS	0.71	(0.49, 1.05)	0.084
0	Reference		
1	6.00	(0.35, 101.57)	0.215
2	2.33	(0.22, 25.25)	0.486
3	1.57	(0.16, 15.67)	0.700
4	0.78	(0.08, 8.04)	0.833
Pre PTH	1.01	(0.99, 1.03)	0.369
Continuous Pod1 PTH	1.49	(1.18, 1.90)	0.001
Pod1 PTH > 5.18 pg/mL	6.31	(2.31, 17.20)	< 0.001
Continuous Pod3 PTH	1.39	(1.17, 1.64)	< 0.001
Pod3 PTH > 5.60 pg/mL	8.06	(3.03, 21.43)	< 0.001
Pre Ca	0.14	(0.00, 14.01)	0.403
Pod1 Ca	3.68	(0.11, 126.12)	0.470
Thyroid pathology			
Carcinoma	Reference		
Goiter	1.95	(0.31, 12.30)	0.477
Continuous Pod7 Mg	5,137,891.30	(3242.36, inf.)	< 0.001
Pod7 Mg > 0.80	11.42	(3.62, 36.02)	< 0.001
Continuous Pod7 P	0.04	(0.01, 0.25)	< 0.001
Pod7 P ≤ 1.51	7.13	(2.47, 20.57)	< 0.001
Continuous Pod7 Ca	112.53	(6.38, 1984.65)	0.001
Pod7 Ca ≥ 2.05	9.77	(2.17, 43.88)	0.003

*TT* total thyroidectomy, *CND* central lymph node dissection, *LND* lateral lymph node dissection, *PGRIS* parathyroid glands remaining in situ, *Pre* preoperative, *Pod* postoperative day, *OR* odds ratios

**Table 4:** Multivariate analysis of factors affecting the Pod7 PTH return to normal

Variables	OR	(95%CI)	*P* value
PGRIS	0.517	(0.290, 0.921)	0.021
Pod1 PTH > 5.18 pg/mL	3.439	(0.968, 12.222)	0.056
Pod3 PTH > 5.60 pg/mL	3.745	(1.078, 13.008)	0.038
Pod7 Mg > 0.80	9.221	(2.374, 35.823)	0.001
Pod7 P ≤ 1.51	4.475	(1.262, 15.864)	0.020

*Pre* preoperative, *Pod* postoperative day, *OR* odds ratios

## Discussion

Hypoparathyroidism is one of the most common complications after total thyroidectomy. Because of the "parathyroid splinting" effect, higher serum calcium concentrations in the first week after thyroid surgery correlate with better recovery of parathyroid function [9]. Lorente-Poch et al. further clarified that maintaining serum calcium at least 9.5 mg/dL after thyroid surgery is beneficial to the recovery of parathyroid function [14]. In agreement with previous findings [9, 14], in the present study, parathyroid function in most patients returned to normal within 1 month (87.39%) after the operation, and intraoperative PGRIS score was an independent influencing factor for the return of normal PTH after surgery. In addition, we found that serum ion concentrations were predictors of parathyroid function return to normal on the seventh postoperative day. Two studies have suggested that intraoperative PGRIS, intraoperative accidental resection, and the number of parathyroid glands that are autotransplanted are not influencing factors for postoperative hypocalcemia [15, 16]. However, the surgical methods in these studies were relatively conservative, and the sample sizes were relatively small and less representative of the population of patients undergoing total thyroidectomy.

Studies have shown that the scope of surgery is a risk factor for hypoparathyroidism after thyroid surgery [17–19]. The greater the scope of the operation, the greater the chance of parathyroid gland parenchyma damage, blood vessel damage, and accidental resection. However, during the recovery of parathyroid function, Thomusch et al. found that intraoperative PGRIS was a more effective variable than the scope of surgery and that the surgical scope was not an independent influencing factor for postoperative recovery of parathyroid function [9, 14]. Our research found similar results. Our results indicate that the effect of the scope of surgery on hypoparathyroidism is not statistically significant. There are two potential explanations for this finding. On the one hand, the scope of the operation may have little effect on parathyroid function return to normal after surgery; on the other hand, most of the patients in this study (108, 96.34%) underwent bilateral total thyroidectomy + CND, and LND could not be performed independently of CND. Parathyroid function recovery is a dynamic process*.* Pod1 PTH and Pod3 PTH represent the baseline level of parathyroid function recovery. Higher Pod1 PTH and Pod3 PTH lead to easier recovery of parathyroid function after surgery. Although the p-value of Pod1 PTH > 5.18 pg/mL was greater than 0.05 (P = 0.056), it was retained in the stepwise forward logistic regression results output by SPSS. Because of its greater influence on the equation, combined with its clinical relevance and impact on the logistic regression model, this variable is still considered meaningful.

During the recovery of parathyroid function, multiple ions work in concert. From a physiological point of view, the decrease in serum magnesium concentration after thyroid surgery inhibits PTH secretion and thus increases the resistance of terminal organs to PTH [20, 21]. PTH increases the reabsorption of magnesium by the renal tubules, and high phosphorus concentrations stimulate the secretion of PTH [22, 23]. Our study also found that parathyroid function recovery to normal after total thyroidectomy was associated with Pod7 Mg and Pod7 P levels. The multivariate analysis results showed that serum magnesium concentration and phosphorus concentration were independent factors influencing the return of Pod7 PTH to normal, and patients with Pod7 Mg > 0.8 mmol/L and Pod7 P ≤ 1.51 mmol/L (P < 0.05) experienced a more rapid return to normal parathyroid function.

Parathyroid hormone is the main hormone regulating calcium ions. Hypocalcemia often occurs after hypoparathyroidism. Many earlier studies reported that the magnesium concentration after thyroid surgery was closely related to hypoparathyroidism [24–27]. We also found that Pod7 Mg and Pod7 P were effective predictors of the return of PTH to normal levels on Pod7. The combined sensitivity of serum Mg and P concentrations to predict normal parathyroid function was 82.76%, and the specificity was 76.83%. Although serum ions are not sufficient to replace PTH, the predictive effect of rapid biochemical indicators on parathyroid function will be helpful for clinicians to diagnose and better manage the dosage of calcium tablets and calcitriol in patients. However, many junior hospitals, such as community hospitals, either do not have the equipment to detect PTH or find that the test is too time-consuming. When patients return to the community hospital, doctors can use serum ions for the early diagnosis of patients with hypocalcemia after thyroid surgery. From the perspective of medical expenses, simultaneous detection of PTH and serum ions can reduce the detection times of PTH in some specific patients (patients with normal PTH values but lower than normal serum ions) and reduce the economic burden of patients. Thus, it is essential to detect ions other than calcium ions after thyroid surgery.

Serum PTH concentration and serum magnesium concentration showed a downward trend within 1–2 days after total thyroidectomy, whereas serum ionized phosphorus showed an upward trend and a gradual return to the preoperative level [27, 28]. Our study further explored the changing trends in postoperative calcium, magnesium, and phosphorus ion concentrations within the first month after surgery. This study also has several limitations related to its retrospective nature. The study protocol required follow-up of patients' recovery of parathyroid function and serum ions within 1, 3, 7, and 30 days and 1 year after surgery. We conservatively estimated that those patients' parathyroid function returned to normal, which may have led to overestimation of parathyroid function recovery. Perhaps because the samples we selected were all patients with hypoparathyroidism, and the samples were special, there were no statistically significant results in BMI, diabetes, operation time or vitamin D. Postoperative preventive calcium and calcitriol supplementation can affect the measurement of real postoperative serum calcium and calcitriol concentrations. The dosage of calcium and calcitriol was not the same in each patient, and active calcium supplementation can also promote the recovery of parathyroid function. We therefore believe it is necessary to design prospective research for further exploration. We also think it could be interesting to evaluate serum ions levels in patients who did not develop postoperative hypoparathyroidism. We wish to finish it in our continued study.

## Conclusion

We found that serum magnesium, phosphorus and PTH concentrations are important influencing factors. Serum ion is an effective predictor of short-term postoperative parathyroid function return to normal. The combination of serum Mg and P concentrations has good sensitivity and specificity in predicting short-term parathyroid function recovery after thyroidectomy.

## Data Availability

The datasets used and/or analyzed during the current study are available from the corresponding author on reasonable request.

## Abbreviations

PTH: Parathyroid hormone
PGRIS: Parathyroid glands remaining in situ
Pod: Postoperative day
Pre: Preoperative
TT: Total thyroidectomy
CND: Central lymph node dissection
LND: Lateral lymph node dissection
ROC: Receiver operating characteristic
AUC: Area under the ROC curve
SE: Standard error
CI: Confidence interval
PPV: Positive predictive value
NPV: Negative predictive value
